# A Novel Kleefstra Syndrome-associated Variant That Affects the Conserved TPL*X* Motif within the Ankyrin Repeat of EHMT1 Leads to Abnormal Protein Folding*

**DOI:** 10.1074/jbc.M116.770545

**Published:** 2017-01-05

**Authors:** Patrick R. Blackburn, Alexander Tischer, Michael T. Zimmermann, Jennifer L. Kemppainen, Sujatha Sastry, Amy E. Knight Johnson, Margot A. Cousin, Nicole J. Boczek, Gavin Oliver, Vinod K. Misra, Ralitza H. Gavrilova, Gwen Lomberk, Matthew Auton, Raul Urrutia, Eric W. Klee

**Affiliations:** From the a Center for Individualized Medicine and; the b Department of Health Science Research, Mayo Clinic, Jacksonville, Florida 32224,; the c Division of Hematology, Departments of Internal Medicine and Biochemistry and Molecular Biology,; the d Department of Health Science Research, Division of Biomedical Statistics and Informatics,; the e Department of Clinical Genomics,; the f Center for Individualized Medicine,; the i Department of Health Science Research,; the j Department of Neurology, and; the k Laboratory of Epigenetics and Chromatin Dynamics, Epigenomics Translational Program, Center for Individualized Medicine, Mayo Clinic, Rochester, Minnesota 55905,; the g Department of Pediatrics, Division of Genetics and Metabolic Disorders, Wayne State University School of Medicine, Detroit, Michigan 48201, and; the h Department of Human Genetics, University of Chicago, Chicago, Illinois 60637

**Keywords:** DNA sequencing, functional genomics, genetic disease, molecular dynamics, molecular modeling, 9q34 deletion syndrome, EHMT1, GLP, Kleefstra syndrome, functional validation

## Abstract

Kleefstra syndrome (KS) (Mendelian Inheritance in Man (MIM) no. 610253), also known as 9q34 deletion syndrome, is an autosomal dominant disorder caused by haploinsufficiency of euchromatic histone methyltransferase-1 (*EHMT1*). The clinical phenotype of KS includes moderate to severe intellectual disability with absent speech, hypotonia, brachycephaly, congenital heart defects, and dysmorphic facial features with hypertelorism, synophrys, macroglossia, protruding tongue, and prognathism. Only a few cases of *de novo* missense mutations in *EHMT1* giving rise to KS have been described. However, some *EHMT1* variants have been described in individuals presenting with autism spectrum disorder or mild intellectual disability, suggesting that the phenotypic spectrum resulting from EHMT1 alterations may be quite broad. In this report, we describe two unrelated patients with complex medical histories consistent with KS in whom next generation sequencing identified the same novel c.2426C>T (p.P809L) missense variant in *EHMT1*. To examine the functional significance of this novel variant, we performed molecular dynamics simulations of the wild type and p.P809L variant, which predicted that the latter would have a propensity to misfold, leading to abnormal histone mark binding. Recombinant EHMT1 p.P809L was also studied using far UV circular dichroism spectroscopy and intrinsic protein fluorescence. These functional studies confirmed the model-based hypotheses and provided evidence for protein misfolding and aberrant target recognition as the underlying pathogenic mechanism for this novel KS-associated variant. This is the first report to suggest that missense variants in EHMT1 that lead to protein misfolding and disrupted histone mark binding can lead to KS.

## Introduction

Kleefstra syndrome (KS)^4^ (Mendelian Inheritance in Man (MIM) no. 610253) is a rare autosomal dominant disorder that is caused by pathogenic variants involving the euchromatic histone methyltransferase-1 (*EHMT1*) gene or subtelomeric deletions of 9q34.3, which include *EHMT1*. Patients with KS typically present with many recognizable clinical features including moderate to severe intellectual disability with absent speech, hypotonia, microbrachycephaly, congenital heart defects, and dysmorphic facial features including hypertelorism, synophrys, macroglossia, protruding tongue, and prognathism (1–3). Over 100 cases have been described in the literature, all with either deletions of or pathogenic variants in *EHMT1* (1–4). Of the currently described cases, most genetic alterations involving *EHMT1* arose *de novo* in the germ line. However, familial cases have been described with maternal mosaicism (5, 6). Clinical severity varies, with some individuals exhibiting only mild intellectual disability and apraxia of speech and others with more severe clinical manifestations, typical of the disorder (7). In addition, there have been reports of individuals with variants in *EHMT1* that present with autism spectrum disorder (ASD) or schizophrenia, suggesting that the phenotypic spectrum may be much broader than is currently appreciated (8, 9).

The *EHMT1* gene encodes a histone lysine methyltransferase (commonly known as G9a-like protein, GLP) that mono- and di-methylates lysine 9 of histone H3 (H3K9me1 and H3K9me2) together with its obligate binding partner, EHMT2 (known as G9a) (10). Both EHMT1 and EHMT2 have large N-terminal domains that include six ankyrin repeats that have been shown to confer binding specificity for H3K9me1 and H3K9me2, as a part of the chromatin reading function of both proteins (10). The EHMT1 ankryin repeat domain has 2-fold greater preference for H3K9me1 compared with EHMT2, whereas the opposite is true for H3K9me2, suggesting the differential binding conferred by each protein results in greater overall affinity for both substrates (10).

Pathogenic variants or deletions in *EHMT1* resulting in haploinsufficiency lead to disease in patients. Studies in heterozygous Ehmt1^+/−^ mice revealed that these animals exhibit reduced exploration and show altered social behavior with measurable deficits in spatial learning and memory compared with wild type animals (11). The *Ehmt1*^+/−^ mice also have structural and functional postsynaptic defects, including significant reductions in spine density, mature spine number, and dendritic arborization, particularly within CA1 hippocampal neurons (12). Additional functional studies of synapses in the CA3-CA1 subfields revealed altered short term plasticity in these animals using the paired pulse facilitation assay (12). Subsequent studies have shown that EHMT1 regulates homeostatic plasticity through control of synaptic scaling (13). Specifically, EHMT1/2 appear to be involved in H3K9me2-mediated transcriptional repression of brain-derived neurotrophic factor during synaptic scaling up in mice, and loss of function mutations in humans are hypothesized to lead to improper neural circuit formation in patients with KS during early development (13). However, the mechanisms underlying abnormal function of pathogenic missense *EHMT1* variants are not well understood.

In the current study, we provide novel insights into the molecular mechanisms associated with the development of KS, through the biochemical characterization of a new EHMT1 protein variant identified in unrelated patients affected by this disease within the setting of a precision medicine clinic. Specifically, we find the alteration of an evolutionarily conserved TPL*X* motif (p.P809L) within the ankyrin repeat affects the structure and dynamics of the protein, thereby impacting its ability to bind its histone mark substrate. Because TPL*X* repeats are present in other regions of the ankyrin repeat, these findings not only bear relevance for understanding the deleterious effects of the p.P809L variant but may help predict the pathogenicity of other variants affecting TPL*X* motifs throughout the protein. The results described herein therefore have both biochemical and biomedical relevance to the diagnosis and mechanistic characterization of KS associated variants.

## Results

### 

#### 

##### Clinical Description and Laboratory Evaluations

The novel EHMT1 variant characterized in this study, was discovered during clinical genetic testing in two unrelated patients that presented with clinical and phenotypic features consistent with KS. Briefly, patient 1 presented with global developmental delay, ASD, aphasia, distinctive craniofacial features, strabismus, an aberrant right subclavian artery, and atrial septal defect. The patient had a normal Canadian newborn screen, karyotype, oligonucleotide array comparative genomic hybridization (aCGH) and SNP (GeneDx) arrays, congenital disorders of glycosylation screen, and plasma/urine amino acids. In addition, *MECP2* sequencing and multiplex ligation-dependent probe amplification (MLPA) testing, Angelman syndrome MS-MLPA, and Fragile X (FMR1 PCR and Southern blot) testing were all normal. The patient was referred to the Mayo Clinic (Rochester, MN) for additional testing. A purine and pyrimidine panel, creatine disorders panel, and Beckwith-Wiedemann syndrome/Russell-Silver syndrome molecular analysis were performed and found to be normal.

Similarly, patient 2 presented with significant intellectual disability, ASD, small size, and dysmorphic features including microbrachycephaly, hypertelorism, synophrys, and prognathia. The patient also had bilaterally increased asymmetric T2 and FLAIR signal in the periventricular and peritrigonal brain regions, with scattered white matter changes on MRI. Patient 2 was initially seen at Children's Hospital of Michigan, where several genetic studies including chromosomal microarray analysis (180K oligo-SNP array) and methylation PCR for Angelman syndrome/Prader-Willi syndrome were performed and found to be normal. Further clinical descriptions of both patients can be found in Table 1.

**TABLE 1:** **Clinical description of the two patients with the p.P809L variant in EHMT1 and correlation with previously reported phenotypes in Kleefstra syndrome** The symbols + and − indicate that the phenotype is present or absent respectively. N/R indicates that the phenotype was not reported or not evaluated.

	Patient 1	Patient 2
Current age (years)	4	16
Sex	Female	Female
Race/ethnicity	White	African American
Height	93.5 cm (∼50th percentile at 3 years)	146 cm (<5th percentile at 14.5 years)
Weight	15.4 kg (∼75th percentile at 3 years)	35.1 kg (<5th percentile at 14.5 years)
Head circumference	49.0 cm (∼60th percentile at 3 years)	53 cm (∼25th percentile at 14.5 years)
Intellectual disability	+	+
Speech delay	+	+
Childhood hypotonia	+	+
Microcephaly	−	+
Short stature	−	+
Overweight	+	−
Brachycephaly	−	+
Midface hypoplasia	−	+
Coarse facies	+	+
Hypertelorism	−	+
Synophrys	−	+
Arched eyebrows	−	−
Short nose	−	−
Anteverted nostrils	−	−
Macroglossia	−	−
Cupid bow upper lip	−	+
Thick/everted lower lip	−	−
Pointed chin	+	−
Dysplastic ear helices	+	−
Brachydactyly	−	−
Cardiac anomaly	+	+
Renal anomaly	N/R	+
Behavioral problems	+	+
Hearing loss	−	−
Seizures	−	−
Brain MRI findings	No structural abnormalities noted; normal MRI	Bilateral increased asymmetric T2 and FLAIR signal in the periventricular and peritrigonal regions and scattered white matter changes in the centrum semi-ovale, which could either be due to gliosis or related to dysmyelination
Additional features	Aberrant right subclavian artery and ASD, tracheomalacia, long tubular epiglottis, tonsillar hypertrophy, sleep apnea, GERD, chronic lung disease, cerebral ataxia, diastasis recti, hypermobility, monocular elevation palsy/deficiency, blue sclera, exotropia of the left eye, sensory processing disorder	Left kidney upper pole defect, left extra numerary nipple, fifth finger clinodactyly noted, reduced extension at elbow, upper and lower extremities are symmetrical, bilateral pes planus, two café-au-lait macules on upper abdomen

Because the differential diagnosis for neurodevelopmental disorders can be quite broad, extensive molecular evaluations were performed in both patients. Subsequently, clinical whole exome sequencing (Baylor College of Medicine Genetic Laboratory) in patient 1 and a comprehensive non-specific intellectual disability sequencing panel (University of Chicago) in patient 2 were pursued to determine a genetic diagnosis. Sequencing revealed several variants including an identical *EHMT1* variant (Chr9(GRCh38): g.137790891C>T, NM_024757.4(EHMT1): c.2426C>T, NP_079033.4: p.P809L) in both individuals (Table 2). The p.P809L *EHMT1* variant was determined to be a *de novo* change in patient 1 (Table 2). The inheritance status of this variant could not be determined in patient 2 because this family was lost to follow-up. The p.P809L variant was not observed in the Exome Aggregation Consortium or in the NHLBI GO Exome Sequencing Project databases (14). Similarly, this variant was also not observed in over 126,216 exomes and 15,137 genomes in the recently released Genome Aggregation Database (gnomAD) (14). According to American College of Medical Genetics and Genomics 2015 guidelines, this variant was classified as a variant of uncertain significance in both patients despite clinical and phenotypic evidence suggestive of pathogenicity (15), which prompted us to initiate the current study to search for potential mechanisms by which the function of this protein may be disrupted. Efforts of this type, which seek to provide a mechanistic insight compatible with pathogenicity for disease-associated variant of uncertain significance, are necessary for advancing the field of precision medicine and aid medical practitioners in the future diagnosis and management of genetic diseases.

**TABLE 2:**
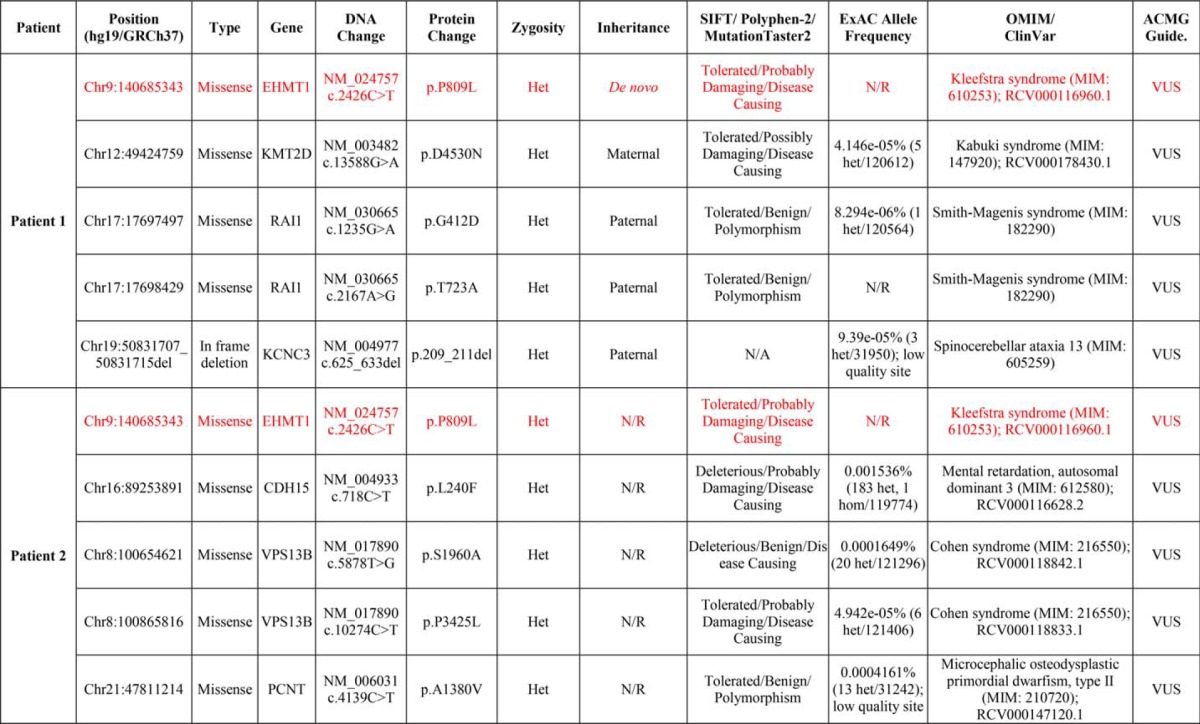
**Summary of clinically reported variants of unknown significance identified in both patients** The EHMT1 p.P809L variant is highlighted in red. VUS, variant of uncertain significance; het, heterozygous; hom, homozygous; N/R, not reported; GERD, gastroesophageal reflux disease.

Clinically, both patients have significant phenotypic overlap with what has been published for KS, including moderate to severe intellectual disability, ASD, global aphasia, and characteristic facial features. There is also variation in their clinical phenotype, including abnormal white matter signal on MRI in patient 2, which has been noted in other cases of KS but which was absent in patient 1 (16). In ∼50% of individuals diagnosed with KS, a heart defect is present. Patient 1 had an atrial septal defect and aberrant right subclavian artery, whereas patient 2 did not have any evidence of a heart defect. Renal defects are also seen in ∼30% of KS patients. Patient 2 had a left upper renal pole defect, but renal ultrasound in patient 1 revealed no defects. Together these studies suggest a possible role for the novel EHMT1 p.P809L variant identified in both patients in disease. Thus, subsequent studies aimed at determining the structural and biochemical impacts underlying the alteration in this protein were undertaken.

##### Structural Analyses Reveal That the p.P809L Variant Likely Alters the Biochemical Properties of EHMT1

**FIGURE 1.**
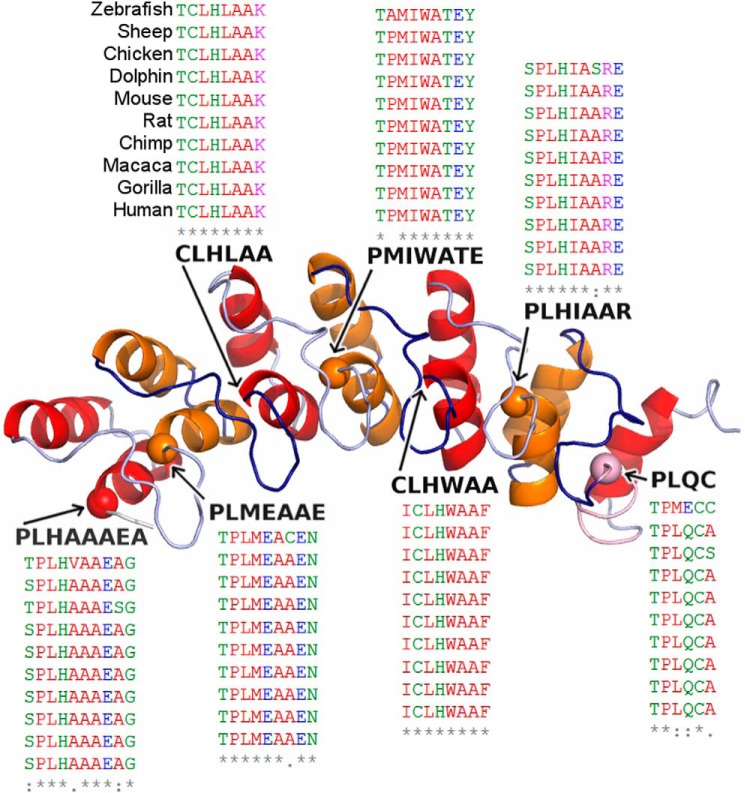
**Diagram of the repeated helix-loop-helix domains that characterize the ankyrin repeat of the EHMT1.** Our model of EHMT1 is shown with repeats colored alternating between *red* and *orange*. Each repeat has an outer and an inner helix. The inner helix always begins with a proline or cysteine and is followed by a conserved TPL*X* motif. The proline α carbons from these motifs are marked with a *sphere*, and the sequence of each motif is shown. The p.P809L falls within the third helix and begins the motif within the second ankyrin repeat. To indicate the conservation of TPL*X* motifs, a section from a multiple sequence alignment of EHMT1 orthologs is shown for each; the species order is shown once for brevity; alignment and corresponding coloring were performed using Clustal Omega (33).

The ankyrin repeat of the EHMT1 protein adopts a helix-loop-helix domain structure. This domain is known to fold cooperatively (17). Cooperative folding indicates that “nucleating” folding events increase the probability of further folding. In three dimensions, the protein domain is organized with an outer layer of longer helices and an inner layer of shorter helices, arranged in anti-parallel fashion and followed by an outward facing loop region. Each repeat is comprised of one long and one short helix. These repeats pack against the helices of the adjacent repeat (Fig. 1) and are stabilized by both hydrobophic interactions and hydrogen bonds (18). Studies of a large number of ankyrin repeats have shown that this domain contains a number of residues that are evolutionarily conserved in organisms ranging from *Drosophila* to humans (Fig. 1) (19). It has been shown that the proline within the TPL*X* motif initiates a tight turn responsible for the helix-turn-helix conformation and contributes to conformational stability of the ankyrin repeat, which is stabilized by hydrogen bonding interactions with other residues within the helix and preceding loop. Thus, based on the conservation (Fig. 1) and contribution of the conserved proline to the proper folding and dynamics of the ankyrin domain, we hypothesized that the substitution of a TPL*X* repeat for TLL*X*, as in the p.P809L variant, alters either the structure and/or dynamics of the protein, thereby impacting its function. Consequently, we tested this hypothesis using molecular modeling and molecular dynamics (MD) simulations, as well as a combination of biophysical methods that compared the structural and dynamic properties of the WT EHMT1 and the p.P809L variant. Initially, we used a series of *in silico* prediction algorithms to determine the potential impact of the p.P809L variant. SIFT, PolyPhen-2, and MutationTaster2 predicted p.P809L to be tolerated, probably damaging, and disease-causing, respectively (Table 2) (20–22). These tools often have conflicting predictions and vary in accuracy and reliability, which prompted us to investigate this variant further using 3D structure-based analyses.

The energy minimized structures were analyzed to better characterize alterations to EHMT1 induced by p.P809L. First, FoldX was used to evaluate the change in folding energy: ΔΔ*G*_fold_ = 3.77 kcal/mol (23). Thus, FoldX predicts p.P809L to be highly destabilizing. Next, we quantified the changes in solvent-accessible surface area (SASA) using NACCESS (24). p.P809L leads to an increase in total SASA (150.2 Å^2^) primarily via greater side chain non-polar SASA (95.7Å^2^); backbone SASA was slightly lower (−18.2 Å^2^) and polar side chain SASA greater (54.5 Å^2^). After alignment with combinatorial extension, the energy minimized structures differed from one another by 3.0 Å root mean square deviation (RMSD) with the largest deviations at the N and C termini and the histone binding loops (25). Finally, we characterized changes in local bonding patterns associated with p.P809L. These changes included a gain of additional hydrogen bonds between p.T801 and p.H778 and between p.D800 and p.A830. Multiple changes (losses and gains) in hydrogen bonding within the loop from p.N798 to p.T801, as well as within the first and third helices, were observed. Together, these data indicate that, at least at a static level, the p.P809L substitution leads to structural and energetic shifts that differ significantly from the WT protein.

##### Molecular Simulations Suggest p.P809L Increases Misfolding Propensity

**FIGURE 2.**
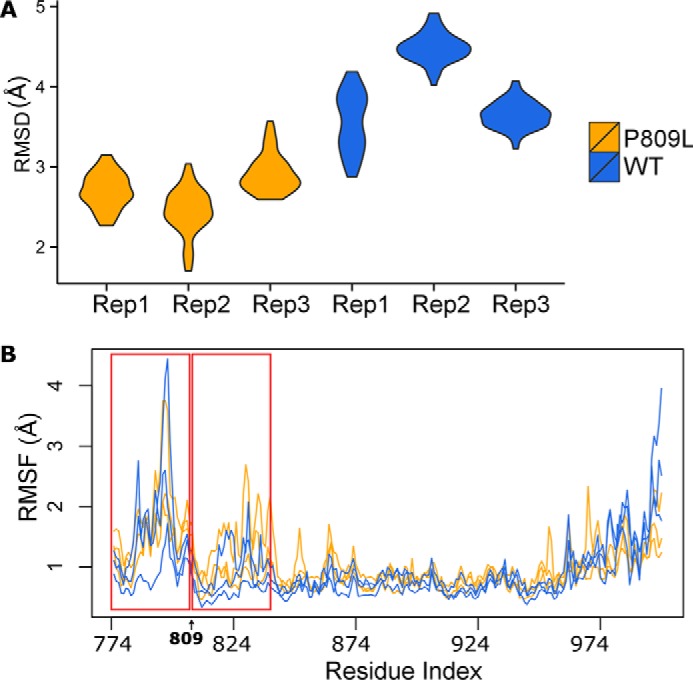
**Molecular dynamic simulations at room temperature.** MD simulations were run in triplicate for 2 and 10 ns with similar results. Data from the 10-ns simulation are shown. *A*, compares simulations by RMSD. *B*, compares changes in RMSF, an indication of the flexibility of the protein during the simulation. The *red windows* highlight the first two ankyrin repeats. RMSF is most strongly affected by p.P809L within these repeats.

To gain insight into the impact of the p.P809L substitution, we performed MD simulations and compared the time-dependent behavior of the WT protein to p.P809L EHMT1. Room temperature (300 K) simulations were analyzed using RMSD and root mean square fluctuation (RMSF) (Fig. 2). RMSD indicates the magnitude of conformational change, whereas RMSF provides an indication of the flexibility of the protein, during the simulation. These measures indicated that p.P809L adopts different conformations than the WT protein and that the second repeat of the ankyrin domain is more mobile as a result of this change. Simulations in the presence of a docked histone tail peptide show that the mutated ankyrin repeat has a conformation more comparable with WT in the presence of substrate (substrate-induced stabilization). Therefore, p.P809L is associated with altered mobility primarily of the first two ankyrin repeats.

**FIGURE 3.**
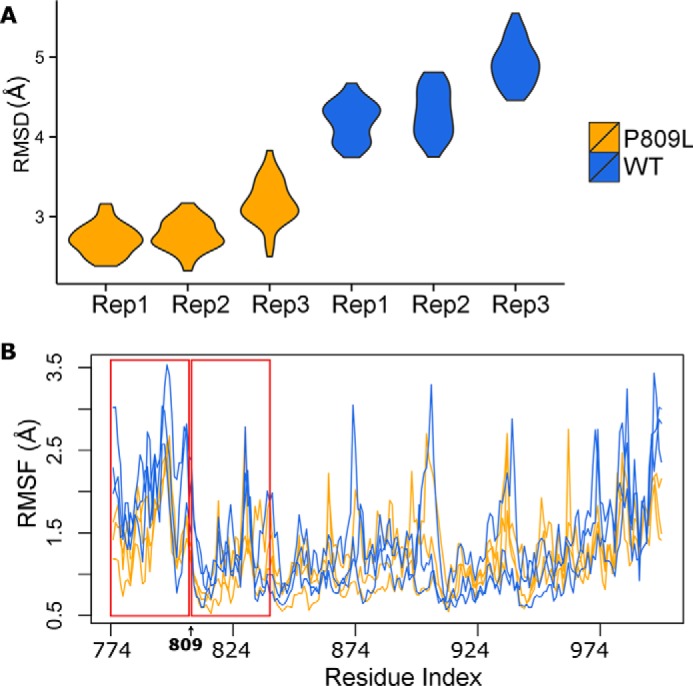
**Molecular dynamic simulations at 360 K.** MD simulations were run for 2 and 10 ns with similar results. Similar to Fig. 2, we compared WT to p.P809L using RMSD (*A*) and RMSF (*B*). The p.P809L variant displays wider changes in RMSD. The *lower panel* shows RMSF as an indication of the flexibility of the protein expressed by the distance that each residue moves during the simulation.

**FIGURE 4.**
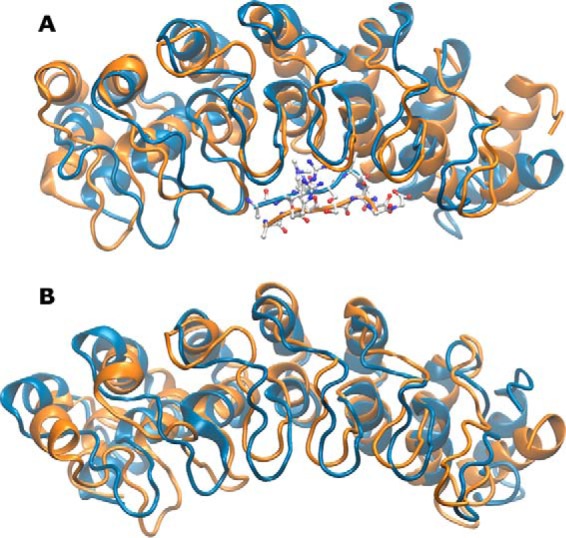
**Mutation leads to structural shifts throughout EHMT1.** The N-terminal ankyrin repeats of the EHMT1 display a high level of structural mobility upon mutation. The final frame from WT (*blue*) and p.P809L (*orange*) simulations and for histone peptide bound (*A*) and apo (*B*) are shown. p.P809 is located at the top of the middle helix of the first ankyrin repeat. Its mutation to leucine results in a shift of the neighboring helix and following loop. These differences propagate through the structure leading to the allosteric conformational change at the C terminus. These changes in the protein are congruent with the previously described cooperative effects of the helix-loop-helix domains for the folding and dynamics of the ankyrin repeat, suggesting that the p.P809L mutation may alter the folding of the protein.

We next performed MD simulations at higher temperature (360 K) that better simulate experimental conditions often used to understand how mutations may impact protein folding. Indeed, this simulation was compared with subsequent experimental denaturation-renaturation data generated at high temperature. These simulations show that the changes in RMSD become even more pronounced at this temperature (Fig. 3). This behavior further indicates that the p.P809L variant likely interferes with protein folding. A comparison of the final adopted conformations in the MD simulations demonstrates a significant distortion of the N-terminal domain of the p.P809L variant as compared with the WT protein (Fig. 4). Thus, molecular simulations indicate a loss of local structural stability.

**FIGURE 5.**
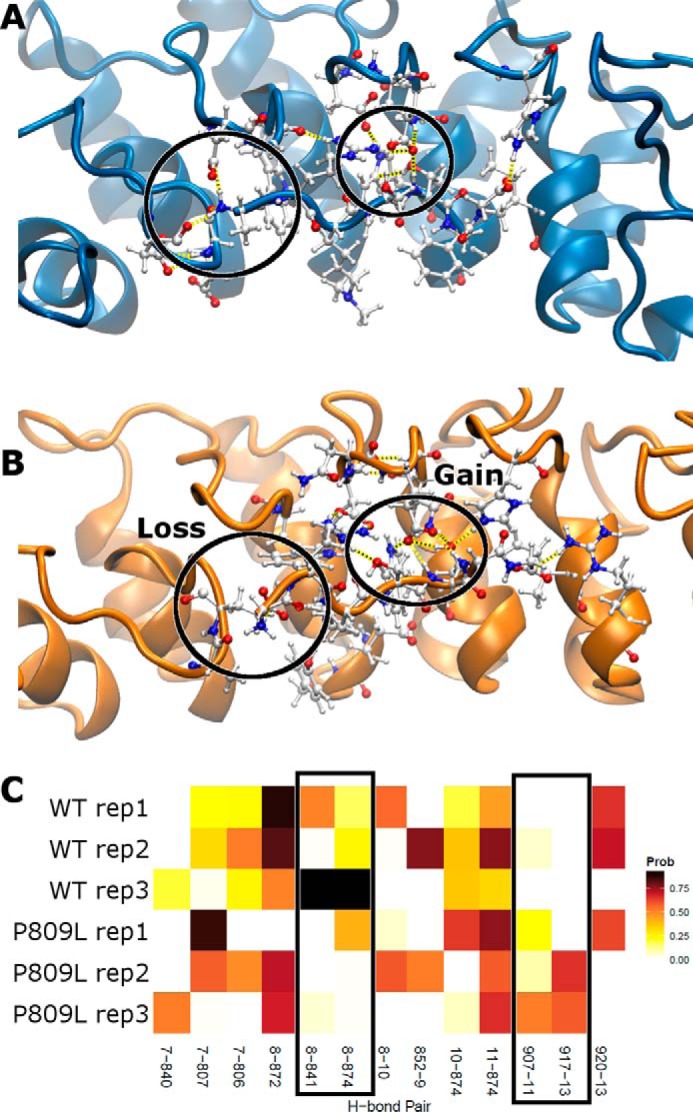
**Altered interactions between EHMT1 and its target.** We show the WT (*A*) and p.P809L (*B*) with residues within and nearby the target peptide shown in detail. Hydrogen bonds are represented as *dashed yellow lines*. Throughout the simulations, the WT and p.P809L demonstrate differences in hydrogen bonding as visible in *A* and *B* and quantified in *C* where the probability of each hydrogen bond interaction is shown. Two pairs are present across WT replicates and lost for p.P809L, whereas two pairs are not present in the WT, but are common in p.P809L (*boxed*). The *abscissa* indicates the residue numbers of interacting residues with the donor residue listed first; residues 7–13 are from the histone tail peptide.

**FIGURE 6.**
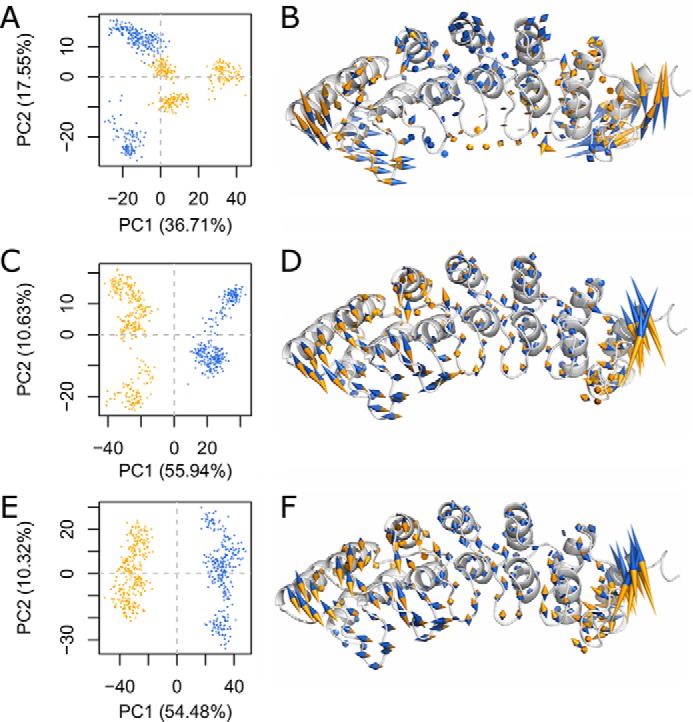
**A single collective motion dominates the differences induced by p. P809L.** We perform PC analysis on the combined trajectories of three different initial conditions with three replicates for each. *A*, *C*, and *E*, each frame from each trajectory is shown as a point in PC space. *B*, *D*, and *F*, the motion indicated by the first PC is shown projected onto the initial WT conformation. The strong anti-correlation between the N and C termini is evident. Because the first PC separates WT from P809L, we colored the motion vectors such that the direction indicates which sequence context (WT, *blue*; p.P809L, *orange*) is represented. *A* and *B*, simulations at 300 K with histone tail peptide bound exhibit a lower degree of motion within the central repeat units, a propensity for the WT to close in around the peptide binding site, and for the p.P809L variant to favor a more extended conformation. *C* and *D*, simulations at 300 K without histone tail peptide demonstrate an opposite trend that is consistent across replicates. *E* and *F*, simulations at 360 K without histone tail peptide are in close agreement with simulations run at 300 K.

Histone tail peptide-bound simulations were performed to analyze hydrogen bonding patterns over time. This analysis was focused on interpeptide hydrogen bonds to more directly indicate how substrate binding may be affected by the variant. We consistently identified residue pairs that gained and lost hydrogen bonding contacts across replicates and over time (Fig. 5). These hydrogen-bonding patterns are known to stabilize the TPL*X* repeat. Overall, the first two and the final repeat of the ankyrin domain displayed similar conformational changes that were consistently observed across our triplicate simulations. Therefore, we conclude that the p.P809L variant in the critical TPL*X* motif impacts bonds that stabilize the typical helix-loop-helix structure of the N-terminal ankyrin repeat domain of EHMT1 and affect the dynamics of the protein. We performed principal component analysis across replicates for each condition to identify and quantify the dominant features within each set of trajectories (Fig. 6). The first PC was a highly dominant motion across all three conditions, representing 37–56% of the total variance sampled. This first PC strongly separated WT from p.P809L simulations. Projecting the first PC's motion onto the initial structure, we identified that in all three conditions, there was correlated motion between the terminal ankyrin repeats that separated WT and p.P809L simulations. When the histone tail peptide was bound, the WT structure tended to maintain contact or establish closer contact with the substrate, whereas p.P809L conformations became more linear. The opposite occurred for apo (no histone tail peptide) simulations at both 300 and 360 K. In summary, our simulation results indicate that the p.P809L variant may increase the misfolding propensity within the N-terminal domain of the protein. Thus, subsequent experiments aimed at testing the folding rates of both proteins using denaturation-renaturation were performed to confirm the results of the molecular dynamic simulations.

##### Biophysical Methods Provide Experimental Validation of the Impact of the p.P809L Substitution on the Folding of the EHMT1 Protein

**FIGURE 7.**
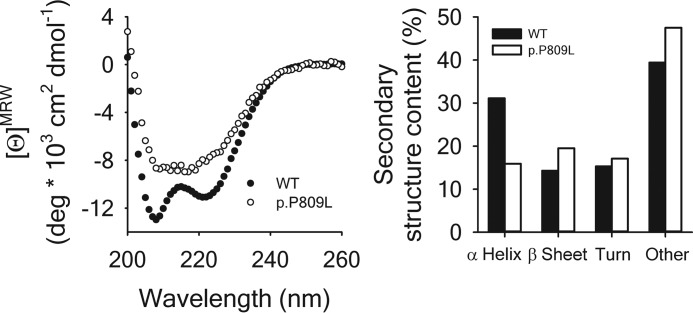
**Impact of the p. P809L mutation on secondary structure.** *Left panel*, far UV CD spectra of WT EHMT1 (●) and of p.P809L (○) were recorded at 20 °C, corrected by the signal of the buffer, and converted to mean ellipticity per amino acid residue (ϴ^MRW^). *Right panel*, deconvoluted secondary structure contents of WT EHMT1 (*solid bars*) and of the p.P809L mutant (*open bars*). Deconvolution of the spectra shown in the *left panel* was performed using the Bestsel software (34).

**FIGURE 8.**
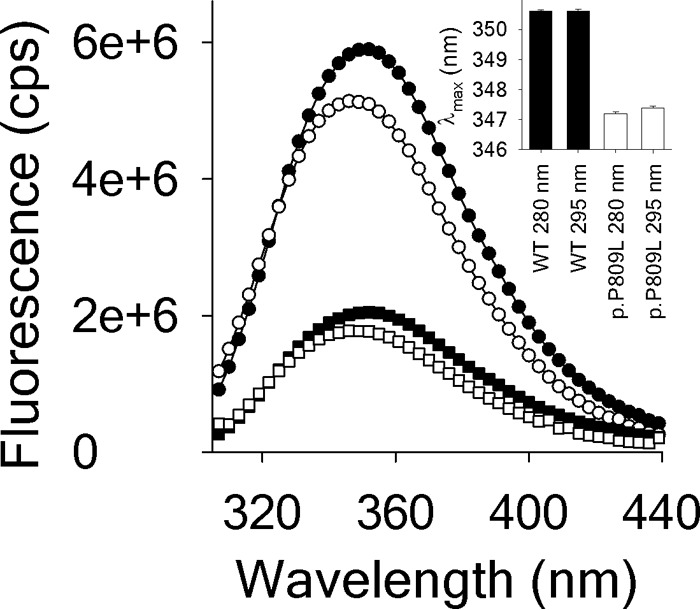
**Fluorescence emission spectra of WT EHMT1 and of p.P809L.** Fluorescence emission spectra of WT EHMT1 (*solid symbols* and *bars*) and of p.P809L (*open symbols* and *bars*) were obtained. Spectra were recorded at 20 °C using a protein concentration of 1 μm. The excitation wavelengths (λ_Ex_) were 280 nm (*circles*; excitation of Trp and Tyr residues) and 295 nm (*squares*; selective excitation of Trp residues). The *inset* illustrates the differences in the wavelength of the maximum intensity (λ_max_).

**FIGURE 9.**
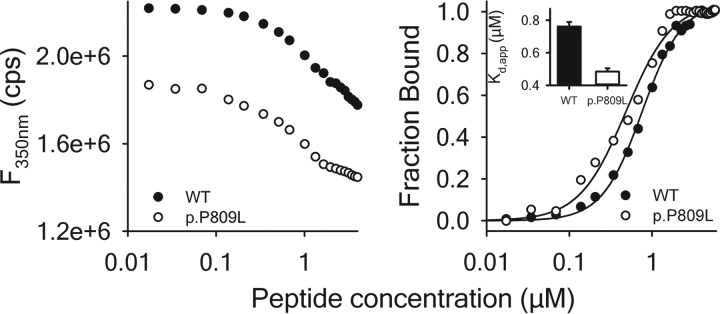
**Interaction of EHMT1 with the dimethylated histone H3 Lys^9^ peptide.** *Left panel*, titration of the peptide into a solution containing 1 μm EHMT1 WT (●) and p.P809L (○). The change in fluorescence intensity was followed at λ_Em_ = 350 nm after excitation at λ_Ex_ = 295 nm. *Right panel*, binding curves for both proteins derived from the data shown in the *left panel*. The *inset* shows the obtained apparent affinities.

Circular dichroism spectra (Fig. 7, *left panel*) were recorded to study the effect of the p.P809L mutation on the secondary structure of EHMT1 and confirm the *in silico* predictions that the p.P809L variant causes abnormal protein folding. The far UV CD spectrum of EHMT1 is dominated by the α-helical content of the protein showing two characteristic minima at 222 and 208 nm. Comparatively, the spectrum of p.P809L EHMT1 shows a reduced α-helical content, which is confirmed by the calculation of secondary structure contributions (Fig. 7, *right panel*). Therefore, in agreement with our molecular simulations, these results demonstrate that the p.P809L mutation leads to an abnormal protein folding behavior of the EHMT1 ankyrin domain. We sought further validation of these results by generating fluorescence emission spectra (Fig. 8) of the WT EHMT1 and the p.P809L variant, at a protein concentration of 1 μm using excitation wavelengths of 280 and 295 nm for the selective excitation of Trp residues, respectively. The wild type protein has a λ_max_ of ∼350 nm, whereas the p.P809L mutation causes a blue shift of the spectrum to 347 nm and a decrease of the fluorescence emission, which is indicative of an altered tertiary structure. Lastly, because the ankyrin repeat domain of the EHMT1 is critical for binding to methylated histones to regulate gene expression, we also studied the interaction of EHMT1 and the p.P809L mutation with its dimethylated target, the histone H3K9 peptide, by monitoring the Trp fluorescence emission at 350 nm after excitation at 295 nm (Fig. 9, *left panel*). The addition of the H3K9me2 peptide causes a quenching of the fluorescence emission indicating an interaction of the peptide with both proteins. Analysis of binding curves shows an apparent affinity of 0.76 μm for WT EHMT1 and 0.48 μm for the p.P809L mutant (Fig. 9, *right panel*). Thus, the p.P809L mutant protein not only displayed loss of secondary structure (unfolding) but also changes in histone reading affinity. In conclusion, molecular modeling and molecular dynamics simulations, when combined with experimental biophysical assays, clearly demonstrate that the p.P809L mutation causes an alteration in the folding and histone reading function of EHMT1.

##### Examination of Non-synonymous Human Population Level Variation within the Ankyrin Repeat Domain of EHMT1 Reveals Greater Amino Acid Conservation within the N-terminal Region Where the p.P809L Variant Falls

**FIGURE 10.**
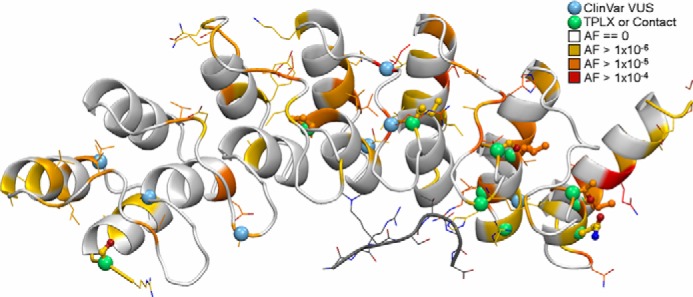
**The interpretation of population and clinically observed variants across the EHMT1 ankyrin repeat domain is aided by structural biology.** We annotated our model by the maximum population allele frequency (*AF*) of variants within each amino acid codon. The coloring is on a log scale where residues with no variation are *white*, *orange* indicates the presence of at least one allele with a missense variant, and *red* indicates the greatest observed allele frequency in gnomAD. *Small green spheres* mark variants in TPL*X* motifs or that interact with the bound histone tail peptide (*gray*). The side chains of any site harboring a missense variant are shown; they are shown thickly for TPL*X* motif variants. Variants of uncertain significance in ClinVar are marked by *small blue spheres*.

By overlaying the frequency of non-synonymous single nucleotide variants within gnomAD on top of the ankyrin repeat domain structure using a color-coded log scale, we are able to show that the C-terminal side of the domain is more polymorphic (Fig. 10). The N-terminal side appears to be more intolerant to non-synonymous variation, which provides further support for the idea that variants located in this region, like the p.P809L reported here, likely affect the structure and/or function of this protein. The sites with the highest variant frequency were found within the loops connecting helices within repeats or were on the solvent exposed “back” side of the protein, suggesting that variation within these regions is more likely to be tolerated. We then looked at additional missense variants reported in ClinVar and show the location of these seven variants using *blue spheres* in an attempt to infer the effect of these variants (Fig. 10). Only one is within a TPL*X* motif (p.P809L). Two of these variants, which have a higher frequency of occurrence, are within the same loop in the middle of the domain. Three of the variants analyzed are packed between helices and could affect stability, though they are mostly conservative amino acid substitutions. The last one is an Ala to Thr at the end of a histone-facing loop (p.A947T) that does not interact with the histone tail peptide in our models, but it could interact when the peptide is analyzed within the context of the full histone protein assembled into a nucleosome. Thus, this analysis provides insights into potential deleterious effects of N-terminal substitutions within EHMT1, setting the stage for future studies focused on determining the role of these variants in disease.

## Discussion

The current study integrates clinical genetic testing, molecular modeling, molecular dynamic simulations, and biochemical studies of protein folding and protein-protein interactions to advance our understanding of the biochemical mechanisms underlying functional alterations in the EHMT1 protein causing KS. Next generation sequencing was used to identify a novel missense variant in *EHMT1* (Chr9(GRCh38): g.137790891C>T, NM_024757.4(EHMT1): c.2426C>T, NP_079033.4: p.P809L) in two unrelated patients with KS. We find that p.P809L falls within an evolutionarily conserved TPL*X* repeat motif of the ankyrin repeat found in many proteins (19). Interestingly, p.P809 falls immediately N-terminal to the beginning of an α helix and stabilizes this secondary structure component of the protein by providing a distinct and necessary angle. This stabilizing aspect is quite different from the destabilizing impact a proline variant often has in an α-helix. Thus, the conservation and topology of the p.P809 within the EHMT1 by itself suggest a leucine substitution would be functionally deleterious to the protein. Given that the Pro to Leu substitution would alter the peptide angle after the adjoining helix and that the helical repeats are cooperative (17), it was likely that this change affects the proper folding and dynamics of EHMT1. Indeed, our molecular modeling and MD simulations support this hypothesis. Molecular dynamic simulations show altered RMSF values for the protein structure when performed at 300 K (26.85 °C, room temperature), with enhanced effects at 360 K (86.85 °C). Moreover, superimposition of the p.P809L with the WT protein structure at the end of the MD simulations clearly illustrate that the N-terminal region of the ankyrin repeat is highly distorted. More importantly, our *in silico* predictions are supported by experimental studies using the WT and p.P809L EHMT1 variant ankyrin domains expressed from *Escherichia coli*. Far UV circular dichroism spectroscopy and intrinsic protein fluorescence were used to evaluate the effect of the p.P809L variant on the structure and the function of the protein. These experiments clearly demonstrate a decrease in the α-helical content in the p.P809L variant when compared with WT. Thus, together, *in silico* studies using stringent molecular mechanics and dynamics algorithms combined with biophysical measurements demonstrate that a TPL*X* to TLL*X* substitution alters the structure and dynamics of the protein. Interestingly, the existence of additional TPL*X* motifs within the ankyrin repeat of the EHMT1 protein suggest that similar alterations in other TPL*X* domains could also result in disrupted protein folding. These results increase our understanding of biochemical mechanisms underlying disease-causing missense variants observed in EHMT1 that fall outside of the catalytic Su(var)3–9, E(z), and Trithorax (SET)domain.

Ankyrin repeats are critical to the ability of EHMT1 to read the H3K9me1 and 2 histone marks. Thus, there was a strong likelihood that the predicted and observed structural and dynamic alterations caused by the p.P809L variant may also alter this histone reading function. MD simulations demonstrate that p.P809L alters the time-dependent interactions of the EHMT1 reader domain with its corresponding histone substrate. We confirmed this effect in Trp-fluorescence quenching experiments using recombinant EHMT1 protein in the presence of the H3K9me2 peptide. These experiments showed altered substrate binding, characterized by a decreased *K_d_* in the p.P809L variant compared with the WT protein. Ultimately, these changes in the interaction of the p.P809L variant with the histone tail peptide reflect alterations in the association and/or dissociation rates of these proteins caused by the structural and dynamic alterations observed in the *in silico* and experimental studies.

Nevertheless, our study is the first to describe how mutations in any of the TPL*X* motifs of the EHMT1 ankyrin domain affect protein folding to induce a moderate change in its affinity for the H3K9me2 peptide. Thus, it becomes important to compare this information to other, better characterized mutations in ankyrin repeats that are known to be pathogenic in humans. Notably, an equivalent p.P81L mutation in the TPL*X* motif within the ankyrin repeat of the tumor suppressor p16, which causes familial melanoma, also leads to secondary structure alterations (26, 27). Thus, this type of mutation (TPL*X* to TLL*X*) is not only destabilizing for EHMT1 but also for other proteins, further supporting the validity of our observations. Notably, however, it is not possible to compare how this change affects the affinity of p16 for its targets because no *K_D_* values have been published for the p.P81L p16 variant. However, it is known that p.P81L p16 displays impaired interaction with CDK4 when measured by a non-quantitative two-hybrid GAL4-based reporter assay (26). Based on these results, we conclude that impaired folding and changes in affinity are likely a shared mechanism underlying the functional alterations of conserved TPXL motifs in the ankyrin repeats of both proteins. These two lines of evidence, together with the fact that TPXL motifs are highly conserved in ankyrin repeats across evolution, raises the possibility that other proteins possessing similar mutations may also be pathogenic. Thus, this knowledge may guide future investigators in the identification and functional characterization of pathogenic variants, which may share a similar mutational event.

EHMT1 is found in a trimeric complex with EHMT2 (G9a) and WIS, which together maintain epigenetic states through human development and also maintain homeostasis in several organs, including the nervous system (28). Studies have been performed in a variety of organisms, from *Drosophila melanogaster* to human cells, which demonstrate that this complex represses gene expression through its ability to read and write the H3K9me2 histone mark (28). Both EHMT1 and EHMT2 bind to the histone code reader proteins, HP1α, HP1β, and HP1γ. These readers recruit the histone methyltransferase, SUVARH1, which converts H3K9me2 into H3K9me3, leading to further transcriptional repression by inducing the formation of heterochromatin via complex formation with DNA methyltransferases (29). Our studies indicate that the p.P809L variant alters the structure, dynamics, and histone binding properties of the EHMT1 protein, suggesting that a sustained alteration in the regulation of gene expression in conserved developmental pathways caused by misfolding and defective substrate binding could similarly give rise to KS in patients.

In conclusion, we have identified a novel p.P809L missense variant in two unrelated patients affected by KS. The p.P809L mutation occurs within a conserved TPL*X* motif that is part of the ankyrin repeat of the EHMT1 protein. Because of the conservation and topology of this substitution, we predicted it may cause alterations in the structure and function of the protein. Through a combination of both *in silico* and experimental techniques, we reveal that the p.P809L variant disrupts the structure and dynamics of the protein in a manner that alters the interaction of this protein with its H3K9 histone tail substrate. Together, these results provide insights into the biochemical mechanisms underlying the function of disease-causing variants involved in the pathogenesis of KS.

## Experimental Procedures

### 

#### 

##### Molecular Identification of a Novel EHMT1 Kleefstra Syndrome-associated Variant

Patient 1 underwent whole exome sequencing (Baylor Miraca Genetics Laboratories) as previously described (30). Briefly, genomic DNA was extracted from the proband, fragmented by sonication, and then ligated to multiplexing paired end adapters (Illumina). The adapter-ligated DNA was then PCR-amplified using primers with sequencing barcodes. For exome capture, the precapture library was enriched by hybridizing to biotin-labeled VCRome 2.1 (Roche NimbleGen) in-solution exome probes at 47 °C for 64–72 h. To improve overall exome coverage, probes to 1800 Mendelian disease genes were also included in the capture. The postcapture DNA library was subjected to massively parallel sequencing on an Illumina HiSeq 2000 platform with 100-bp paired end reads. On average over 70% of reads aligned to target, more than 95% of target bases have greater than 20× coverage, more than 85% of target bases have greater than 40× coverage, and the overall mean coverage of target bases is greater than 100×. The output data from Illumina HiSeq was converted to FastQ file by CASAVA 1.8 (Illumina) and mapped using the Burrows-Wheeler Alignment tool to the Genome Reference Consortium human genome build 37, human genome 19 (GRCh37/hg19). The variant calls were performed using Atlas-SNP and Atlas-indel developed by the Baylor College of Medicine Human Genome Sequencing Center. The variants were annotated using HGSC-SNP-anno and HGSC-indel-anno (Baylor College of Medicine Human Genome Sequencing Center). Variants were then compared with reported mutations from the professional version of the Human Gene Mutation Database. Variants in this database with a minor allele frequency of less than 5% according to either the 1000 Genomes Project or the ESP5400 data of the National Heart, Lung, and Blood Institute GO Exome Sequencing Project were kept. Synonymous variants, intronic variants greater than 5 bp from exon boundaries, and common benign variants (minor allele frequency, greater than 1%) were excluded unless they were reported as pathogenic by Human Gene Mutation Database. The variants were interpreted according to American College of Medical Genetics and Genomics guidelines and patient phenotypes. Variants of interest were then confirmed by Sanger sequencing in both the proband and parental samples to determine inheritance.

Patient 2 was tested using the Comprehensive Non-Syndromic Intellectual Disability 144 Gene Panel (University of Chicago). Briefly, genomic coordinates were identified for all target regions in 144 genes related to a collection of intellectual disability-associated conditions. A custom Agilent SureSelect capture kit (Agilent Technologies, Santa Clara, CA) was used to target the coding sequence plus 10-bp flanking intronic or UTR sequence in the target genes. Sequencing was performed using MiSeq technology with 150-bp paired end reads. Fastq files were aligned using the UCSC human genome build hg19 as a reference. Variants within exons and the 10-bp flanking intronic regions within the 144 target genes were identified and evaluated using a validated, custom bioinformatic pipeline and interpreted by a team of board-certified Ph.D. geneticists and genetic counselors. Gaps or regions of poor coverage in the next generation data set were filled by Sanger sequencing. All novel and likely pathogenic variants on this 144-gene panel were confirmed by Sanger sequencing in the proband. The sensitivity of this test is estimated to be greater than 99% for single base changes and for insertions and deletions of less than 20 bp.

##### Molecular Modeling and Molecular Dynamic Simulations Studies on the p.P809L EHMT1 Variant

Molecular models of wild type EHMT1 solved by X-ray crystallography (Protein Data Bank code 3B95) and the p.P809L variant generated using *in silico* mutagenesis, as previously described, were analyzed using MD simulation (31). Simulations of the EHMT1 variants were performed using the all-atom force field in CHARMm c36b2 at temperatures of 300 and 360 K (constant number of particles, volume, and temperature ensemble) (32). The molecule was first energy-minimized using a two-step protocol of steepest descent and conjugated gradients. The SHAKE procedure was used in all stages (31). A distance-dependent dielectrics implicit solvent model was used with a dielectric constant of 80 and a pH of 7.4. Trajectories were run for 10 ns. Independent duplicate trajectories were run for 2 ns to assess the stability and consistency of each simulation. System setup was performed in Discovery Studio (35). Multiple analyses were performed in the R programming language (36), leveraging the bio3d (37) package version 2.2.4. Molecular visualizations were generated using PyMOL (38) version 1.7.6 and VMD (39) version 1.9.2. Trajectories were compared using RMSD, RMSF, and principal component (PC) analysis in Cartesian space, using C^α^ atoms aligned to the initial WT conformation.

##### Generation, Purification, Circular Dichroism, and Fluorescence Emission Spectroscopy Analyses of the p.P809L EHMT1 Variant

For experimental purposes, we produced and purified an N-terminal His_6_-tagged recombinant form of WT and the p.P809L EHMT1 proteins using the pET vector system (Novagen). The plasmids were grown in DE3 BL21 bacteria cells overnight and induced with 0.5 mm isopropyl β-d-thiogalactopyranoside for 90 min at 32 °C. The recombinant proteins were purified using the Thermo Scientific HisPur cobalt resin kit according to the manufacturer's instructions. Protein was dialyzed overnight and concentrated to a final concentration of 1 mg/ml. Spectra in the far UV range (200–260 nm) for WT and p.P809L EHMT1 were recorded on an Aviv Biomedical Model 420SF circular dichroism spectrometer. The path length of the quartz cuvette was 1 mm, the stepwidth and bandwidth were 1 nm, and the integration time was 20 s. The spectra were corrected by the CD signal of the corresponding buffer and converted to molar ellipticity per amino acid residue (ϴ^MRW^). Fluorescence measurements were performed at 20 °C on a Horiba Jobin-Yvon Fluorolog 3 spectrofluorometer equipped with a Wavelength Electronics LF1-3751 temperature controller. Fluorescence emission spectra of both proteins were recorded between 310 and 440 nm after excitation at 280 or 295 nm using a protein concentration of 1 μm in a 1-cm quartz cell. The titration of the dimethylated histone H3 Lys^9^ peptide was performed by stepwise addition of the peptide (36.3 μm) to a solution containing 1 μm of WT or p.P809L EHMT1 under slight stirring. After equilibration for 2 min, the fluorescence signal was recorded and averaged for 20 s using an excitation wavelength of 295 nm for the selective excitation of Trp residues and an emission wavelength of 350 nm. Apparent affinities were determined after normalization to fraction bound using a logistic function: *f* = a*x^b^/(*c*^b^ + *x*^b^), where *a* is to the maximum asymptote, *b* is the Hill slope, and *c* is the midpoint of the curve that is reported as apparent affinity.

## Author Contributions

P. R. B., A. T., M. A., G. A. L., E. W. K., and R. A. U. designed the study. P. R. B., A. T., M. T. Z., J. L. K., S. S., A. E. K., M. A. C., N. J. B., G. O., V. K. M., R. H. G., G. A. L., M. A., E. W. K., and R. A. U. gathered the data. P. R. B., A. T., M. T. Z., G. O., M. A., E. W. K., G. A. L., and R. A. U. analyzed the data. P. R. B., A. T., M. T. Z., G. O., M. A., E. W. K., and R. A. U. wrote the paper.

## Footnotes

4 The abbreviations used are:

KS: Kleefstra syndrome
MD: molecular dynamics
ASD: autism spectrum disorder
gnomAD: Genome Aggregation Database
SASA: solvent-accessible surface area
RMSD: root mean square deviation
RMSF: root mean square fluctuation
PC: principal component
MLPA: multiplex ligation-dependent probe amplification.

## References

[1] Kleefstra T., Kramer J. M., Neveling K., Willemsen M. H., Koemans T. S., Vissers L. E., Wissink-Lindhout W., Fenckova M., van den Akker W. M., Kasri N. N., Nillesen W. M., Prescott T., Clark R. D., Devriendt K., van Reeuwijk J., et al (2012) Disruption of an EHMT1-associated chromatin-modification module causes intellectual disability. Am. J. Hum. Genet. 91, 73–82 22726846 10.1016/j.ajhg.2012.05.003 PMC3397275

[2] Kleefstra T., van Zelst-Stams W. A., Nillesen W. M., Cormier-Daire V., Houge G., Foulds N., van Dooren M., Willemsen M. H., Pfundt R., Turner A., Wilson M., McGaughran J., Rauch A., Zenker M., Adam M. P., et al (2009) Further clinical and molecular delineation of the 9q subtelomeric deletion syndrome supports a major contribution of EHMT1 haploinsufficiency to the core phenotype. J. Med. Genet. 46, 598–606 19264732 10.1136/jmg.2008.062950

[3] Kleefstra T., Brunner H. G., Amiel J., Oudakker A. R., Nillesen W. M., Magee A., Geneviève D., Cormier-Daire V., van Esch H., Fryns J.-P., Hamel B. C., Sistermans E. A., de Vries B. B., and van Bokhoven H. (2006) Loss-of-function mutations in euchromatin histone methyl transferase 1 (EHMT1) cause the 9q34 subtelomeric deletion syndrome. Am. J. Hum. Genet. 79, 370–377 16826528 10.1086/505693 PMC1559478

[4] Willemsen M. H., Vulto-van Silfhout A. T., Nillesen W. M., Wissink-Lindhout W. M., van Bokhoven H., Philip N., Berry-Kravis E. M., Kini U., van Ravenswaaij-Arts C. M., Delle Chiaie B., Innes A. M., Houge G., Kosonen T., Cremer K., Fannemel M., et al (2012) Update on Kleefstra syndrome. Mol. Syndromol. 2, 202–212 22670141 10.1159/000335648 PMC3366700

[5] Willemsen M. H., Beunders G., Callaghan M., de Leeuw N., Nillesen W. M., Yntema H. G., van Hagen J. M., Nieuwint A. W., Morrison N., Keijzers-Vloet S. T., Hoischen A., Brunner H. G., Tolmie J., and Kleefstra T. (2011) Familial Kleefstra syndrome due to maternal somatic mosaicism for interstitial 9q34.3 microdeletions. Clin. Genet. 80, 31–38 21204793 10.1111/j.1399-0004.2010.01607.x

[6] Rump A., Hildebrand L., Tzschach A., Ullmann R., Schrock E., and Mitter D. (2013) A mosaic maternal splice donor mutation in the EHMT1 gene leads to aberrant transcripts and to Kleefstra syndrome in the offspring. Eur. J. Hum. Genet. 21, 887–890 23232695 10.1038/ejhg.2012.267 PMC3722677

[7] Samango-Sprouse C., Lawson P., Sprouse C., Stapleton E., Sadeghin T., and Gropman A. (2016) Expanding the phenotypic profile of Kleefstra syndrome: a female with low-average intelligence and childhood apraxia of speech. Am. J. Med. Genet. A 170A, 1312–1316 26833960 10.1002/ajmg.a.37575

[8] Kirov G., Pocklington A. J., Holmans P., Ivanov D., Ikeda M., Ruderfer D., Moran J., Chambert K., Toncheva D., Georgieva L., Grozeva D., Fjodorova M., Wollerton R., Rees E., Nikolov I., et al (2012) *De novo* CNV analysis implicates specific abnormalities of postsynaptic signalling complexes in the pathogenesis of schizophrenia. Mol. Psychiatry 17, 142–153 22083728 10.1038/mp.2011.154 PMC3603134

[9] Talkowski M. E., Rosenfeld J. A., Blumenthal I., Pillalamarri V., Chiang C., Heilbut A., Ernst C., Hanscom C., Rossin E., Lindgren A. M., Pereira S., Ruderfer D., Kirby A., Ripke S., Harris D. J., et al (2012) Sequencing chromosomal abnormalities reveals neurodevelopmental loci that confer risk across diagnostic boundaries. Cell 149, 525–537 22521361 10.1016/j.cell.2012.03.028 PMC3340505

[10] Collins R. E., Northrop J. P., Horton J. R., Lee D. Y., Zhang X., Stallcup M. R., and Cheng X. (2008) The ankyrin repeats of G9a and GLP histone methyltransferases are mono- and dimethyllysine binding modules. Nat. Struct. Mol. Biol. 15, 245–250 18264113 10.1038/nsmb.1384 PMC2586904

[11] Balemans M. C., Huibers M. M., Eikelenboom N. W., Kuipers A. J., van Summeren R. C., Pijpers M. M, Tachibana M., Shinkai Y., van Bokhoven H., and Van der Zee C. E. (2010) Reduced exploration, increased anxiety, and altered social behavior: autistic-like features of euchromatin histone methyltransferase 1 heterozygous knockout mice. Behav. Brain Res. 208, 47–55 19896504 10.1016/j.bbr.2009.11.008

[12] Balemans M. C., Kasri N. N., Kopanitsa M. V., Afinowi N. O., Ramakers G., Peters T. A., Beynon A. J., Janssen S. M., van Summeren R. C., Eeftens J. M., Eikelenboom N., Benevento M., Tachibana M., Shinkai Y., Kleefstra T., et al (2013) Hippocampal dysfunction in the euchromatin histone methyltransferase 1 heterozygous knockout mouse model for Kleefstra syndrome. Hum. Mol. Genet. 22, 852–866 23175442 10.1093/hmg/dds490

[13] Benevento M., Iacono G., Selten M., Ba W., Oudakker A., Frega M., Keller J., Mancini R., Lewerissa E., Kleefstra T., Stunnenberg H. G., Zhou H., van Bokhoven H., and Nadif Kasri N. (2016) Histone methylation by the Kleefstra syndrome protein EHMT1 mediates homeostatic synaptic scaling. Neuron 91, 341–355 27373831 10.1016/j.neuron.2016.06.003

[14] Lek M., Karczewski K. J., Minikel E. V., Samocha K. E., Banks E., Fennell T., O'Donnell-Luria A. H., Ware J. S., Hill A. J., Cummings B. B., Tukiainen T., Birnbaum D. P., Kosmicki J. A., Duncan L. E., Estrada K., et al (2016) Exome Aggregation Consortium: analysis of protein-coding genetic variation in 60,706 humans. Nature. 536, 285–291 27535533 10.1038/nature19057 PMC5018207

[15] Richards S., Aziz N., Bale S., Bick D., Das S., Gastier-Foster J., Grody W. W., Hegde M., Lyon E., Spector E., Voelkerding K., Rehm H. L. (2015) ACMGLaboratory Quality Assurance Committee: Standards and Guidelines for the Interpretation of Sequence Variants: A Joint Consensus Recommendation of the American College of Medical Genetics and Genomics and the Association for Molecular Pathology. Genet. Med. 17, 405–423 25741868 10.1038/gim.2015.30 PMC4544753

[16] He X., Caluseriu O., Srivastava R., Denny A. M., and Bolduc F. V. (2016) Reversible white matter lesions associated with mutant EHMT1 and Kleefstra syndrome. Neurol. Genet. 2, e58 27123477 10.1212/NXG.0000000000000058 PMC4830196

[17] Barrick D., Ferreiro D. U., and Komives E. A. (2008) Folding landscapes of ankyrin repeat proteins: experiments meet theory. Curr. Opin. Struct. Biol. 18, 27–34 18243686 10.1016/j.sbi.2007.12.004 PMC2680087

[18] Mosavi L. K., Minor D. L. Jr., and Peng Z.-Y. (2002) Consensus-derived structural determinants of the ankyrin repeat motif. Proc. Natl. Acad. Sci. U.S.A. 99, 16029–16034 12461176 10.1073/pnas.252537899 PMC138559

[19] Mosavi L. K., Cammett T. J., Desrosiers D. C., and Peng Z.-Y. (2004) The ankyrin repeat as molecular architecture for protein recognition. Protein Sci. 13, 1435–1448 15152081 10.1110/ps.03554604 PMC2279977

[20] Kumar P., Henikoff S., and Ng P. C. (2009) Predicting the effects of coding non-synonymous variants on protein function using the SIFT algorithm. Nat. Protoc. 4, 1073–1081 19561590 10.1038/nprot.2009.86

[21] Kircher M., Witten D. M., Jain P., O'Roak B. J., Cooper G. M., and Shendure J. (2014) A general framework for estimating the relative pathogenicity of human genetic variants. Nat. Genet. 46, 310–315 24487276 10.1038/ng.2892 PMC3992975

[22] Schwarz J. M., Cooper D. N., Schuelke M., and Seelow D. (2014) MutationTaster2: mutation prediction for the deep-sequencing age. Nat. Methods 11, 361–362 24681721 10.1038/nmeth.2890

[23] Schymkowitz J., Borg J., Stricher F., Nys R., Rousseau F., and Serrano L. (2005) The FoldX web server: an online force field. Nucleic Acids Res. 33, W382–W388 15980494 10.1093/nar/gki387 PMC1160148

[24] Hubbard S. J., and Thornton J. M. (1993) NACCESS, Elsevier

[25] Shindyalov I. N., and Bourne P. E. (1998) Protein structure alignment by incremental combinatorial extension (CE) of the optimal path. Protein Eng. 11, 739–747 9796821 10.1093/protein/11.9.739

[26] Zhang B., and Peng Z.-Y. (2002) Structural consequences of tumor-derived mutations in p16INK4a probed by limited proteolysis. Biochemistry 41, 6293–6302 12009890 10.1021/bi0117100

[27] Cammett T. J., Luo L., and Peng Z.-Y. (2003) Design and characterization of a hyperstable p16INK4a that restores Cdk4 binding activity when combined with oncogenic mutations. J. Mol. Biol. 327, 285–297 12614625 10.1016/s0022-2836(03)00043-3

[28] Shinkai Y., and Tachibana M. (2011) H3K9 methyltransferase G9a and the related molecule GLP. Genes Dev. 25, 781–788 21498567 10.1101/gad.2027411 PMC3078703

[29] Lomberk G., Wallrath L., and Urrutia R. (2006) The heterochromatin protein 1 family. Genome Biol. 7, 228 17224041 10.1186/gb-2006-7-7-228 PMC1779566

[30] Yang Y., Muzny D. M., Reid J. G., Bainbridge M. N., Willis A., Ward P. A., Braxton A., Beuten J., Xia F., Niu Z., Hardison M., Person R., Bekheirnia M. R., Leduc M. S., Kirby A., et al (2013) Clinical whole-exome sequencing for the diagnosis of mendelian disorders. N. Engl. J. Med. 369, 1502–1511 24088041 10.1056/NEJMoa1306555 PMC4211433

[31] Urrutia R., Velez G., Lin M., Lomberk G., Neira J. L., and Iovanna J. (2014) Evidence supporting the existence of a NUPR1-like family of helix-loop-helix chromatin proteins related to, yet distinct from, AT hook-containing HMG proteins. J. Mol. Model. 20, 2357 25056123 10.1007/s00894-014-2357-7 PMC4139591

[32] Velez G., Lin M., Christensen T., Faubion W. A., Lomberk G., and Urrutia R. (2016) Evidence supporting a critical contribution of intrinsically disordered regions to the biochemical behavior of full-length human HP1γ. J. Mol. Model. 22, 12 26680990 10.1007/s00894-015-2874-z PMC4683166

[33] Sievers F., Wilm A., Dineen D., Gibson T. J., Karplus K., Li W., Lopez R., McWilliam H., Remmert M., Söding J., Thompson J. D., and Higgins D. G. (2011) Fast, scalable generation of high-quality protein multiple sequence alignments using Clustal Omega. Mol. Syst. Biol. 7, 539–539 21988835 10.1038/msb.2011.75 PMC3261699

[34] Micsonai A., Wien F., Kernya L., Lee Y.-H., Goto Y., Réfrégiers M., and Kardos J. (2015) Accurate secondary structure prediction and fold recognition for circular dichroism spectroscopy. Proc. Natl. Acad. Sci. U.S.A. 112, E3095–E3103 26038575 10.1073/pnas.1500851112 PMC4475991

[35] BIOVIA (2017) Dassault Systèmes BIOVIA, Discovery Studio Modeling Environment, Release 2017, Dassault Systèmes, San Diego, CA

[36] R Core Team (2014) R: A Language and Environment for Statistical Computing, R Foundation for Statistical Computing

[37] Grant B. J., Rodrigues A. P., ElSawy K. M., McCammon J. A., and Caves L. S. (2006) Bio3d: an R package for the comparative analysis of protein structures. Bioinformatics 22, 2695–2696 16940322 10.1093/bioinformatics/btl461

[38] DeLano W. L. (2012) The PyMOL Molecular Graphics System, Version 1.5.0.3 Schrödinger, LLC, New York

[39] Humphrey W., Dalke A., and Schulten K. (1996) VMD: visual molecular dynamics. J. Mol. Graph. 14, 33–38, 27–28 8744570 10.1016/0263-7855(96)00018-5

